# First detection of rabies virus in encephalitic goats (*Capra hircus*) from Sarawak, Malaysian Borneo: a case study report

**DOI:** 10.1186/s12917-025-05110-2

**Published:** 2025-11-06

**Authors:** Kiing Aik Wong, Amina binti Rusli, George anak Bobby, Michael Woon Hock Lim, Jackie anak Peter, Naziah binti Muntil, Adrian Susin Ambud, David Perera

**Affiliations:** 1 Institute of Health and Community Medicine (IHCM), Universiti Malaysia Sarawak, Kota Samarahan, Sarawak, 94300 Malaysia; 2 Sarawak Veterinary Diagnostic Laboratory, Department of Veterinary Services Sarawak (DVSS), Kota Samarahan, Sarawak, 94300 Malaysia

**Keywords:** Rabies, Goat, Sarawak, DFAT, RIDT

## Abstract

**Background:**

Effective surveillance is essential for detecting rabies virus (RABV) spillover into non-canid mammalian species, which represents an emerging concern for public and veterinary health. This report describes the first documented cases of dog associated rabies in goats in Bintulu, Sarawak, Malaysia, highlighting the critical implications for veterinary and public health.

**Case presentation:**

Two goats were reported to exhibit clinical signs consistent with rabies, including recumbency, hypersalivation, stiffness, dehydration, and episodes of pedalling movements and opisthotonos. Both animals experienced rapid clinical deterioration, leading to their death. The epidemiological investigation found that they had been in contact with a domestic dog displaying neurological signs consistent with rabies and no evidence of rabies vaccination. However, laboratory confirmation of RABV was not possible due to the advanced decomposition of the dog carcass. The rapid immunochromatographic diagnostic test (RIDT) and Direct Fluorescent Antibody Test (DFAT) analyses confirmed the presence of RABV antigen in the brain tissues of both goats. Phylogenetic analysis of RABV sequences obtained from the brain tissue by Next Generation Sequencing (NGS) revealed that the isolates were closely related to previous dog-associated RABV isolates from Sarawak. These findings support the diagnosis and suggest a likely transmission link to the suspected rabid dog present on the farm.

**Conclusion:**

This study underscores the critical need for enhanced rabies control strategies, including widespread vaccination of dogs and rigorous surveillance. The genetic similarity between the RABV detected in goats and those previously detected in dogs and cats in Sarawak suggests a persistent rabies transmission cycle in the region. As such, ongoing surveillance and preventive efforts remain essential to prevent outbreaks and protect both animal and human health.

**Supplementary Information:**

The online version contains supplementary material available at 10.1186/s12917-025-05110-2.

## Background

Rabies is a fatal zoonotic disease caused by the rabies virus (RABV), a member of the *Lyssavirus* genus in the *Rhabdoviridae* family. It primarily affects mammals and is transmitted via the saliva of infected animals, most commonly through bites. Once clinical signs appear, rabies is almost invariably fatal, posing a serious public health threat in endemic regions [1].

In Malaysia, rabies is a notifiable disease under the Prevention and Control of Infectious Diseases Act 1988. Although Peninsular Malaysia has remained largely free from rabies for decades, Sarawak, located on the island of Borneo, has experienced a re-emergence of the disease since July 2017. The state has since reported numerous human fatalities, all linked to dog bites, and persistent rabies activity in domestic and stray animal populations. Dogs (*Canis lupus familiaris*) remain the principal reservoir, with cats (*Felis catus*) acting as secondary hosts [2].

Between 2017 and late 2024, Sarawak recorded a total of 1,140 animal rabies cases from 4,858 samples tested. In 2024 alone, 123 animal rabies cases were confirmed, primarily involving free-roaming dogs across divisions such as Kuching, Bau, Padawan, Samarahan and Bintulu. These statistics reflect the ongoing enzootic status of rabies in Sarawak’s domestic and stray animal populations [3]. 

Rabies infection in livestock, including goats, has been documented in various endemic regions as a result of spillover from rabies-infected dogs [4]. In Malaysia, however, there has been no prior laboratory-confirmed case of rabies in goats reported in the scientific literature. This case report documents the first confirmed RABV infection in a goat in Sarawak, Malaysia. The detection of rabies in this atypical host species underscores the importance of expanding rabies surveillance to include all susceptible animals and reinforces the need for a One Health approach to rabies control.

## Case presentation

In this report, we present two confirmed goat rabies cases identified as SVDL 1959/23 and SVDL 2000/23 from the same farm in Bintulu, Sarawak. While both cases exhibited similar neurological and behavioural signs consistent with rabies infection, they differed in the onset of clinical signs and the circumstances surrounding detection. The clinical signs observed were more consistent with the dumb/paralytic form of rabies. The first case (SVDL 1959/23) involved a 1-year-old female local mix goat showing aggression, excessive bleating, and hypersalivation before progressing to recumbency. The second case (SVDL 2000/23), detected five days later during a follow-up vaccination visit, involved a 1-year-old female Boer cross goat displaying comparable signs but with additional tachycardia and episodes of opisthotonos. A summary of the clinical and epidemiological details is provided in Supplementary Table S1.

### Case 1: SVDL 1959/23

**Fig. 1:**
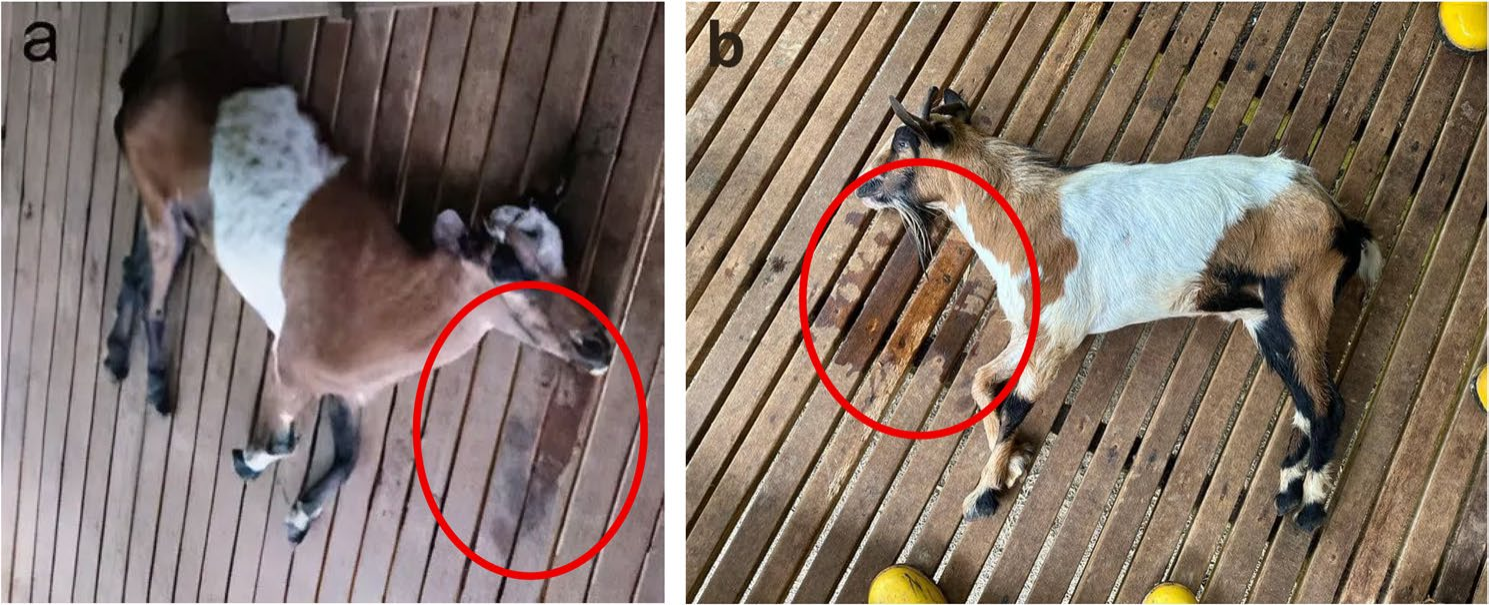
Goats infected by RABV. **A** Case 1 and (**B**) Case 2. Both goats showed typical rabies clinical signs: Hypersalivation (saliva circle in red) and opisthotonos (hindlimb rigidity)

On 21 st September 2023, the Bintulu Veterinary Office, Department of Veterinary Services Sarawak received a complaint from a local meat-producing goat farm in Bintulu regarding a 1-year-old female goat (local mix) (Fig. 1A), exhibiting neurological clinical signs including aggression, excessive bleating, and hypersalivation. During the farm investigation, the Veterinary Officer observed total recumbency, dehydration, excessive drooling, stiffness, and pedalling movements. However, no apparent skin lesions were observed. The goat died naturally 6 h after recumbency, shortly after the examination by the Veterinary Officer.

The Veterinary Officer was informed by the farmer that the goat started to show these clinical signs on the 19th of September 2023. The farmer did not recognize the potential rabies significance of the goat’s clinical signs and, consequently, did not implement isolation measures. During necropsy, the goat was carefully examined for bite wounds or skin lesions; however, none were observed. According to the farm worker, the goat had been bitten on the nose by a suspected rabid dog owned by the farm owner, which was unvaccinated against rabies and allowed to mix freely with the goats, approximately 3 to 7 days before clinical signs appeared on 19 September 2023. Before the incident, all 31 goats had been vaccinated against Pasteurellosis only, with no anti-rabies vaccination administered, as rabies vaccination in goats is not mandated under current state legislation. Based on the clinical signs observed, the differential diagnoses considered were rabies, tetanus, and toxicosis. Given the ongoing canine rabies cases in Bintulu since 2018, the dog likely became infected through local transmission.

**Fig. 2:**
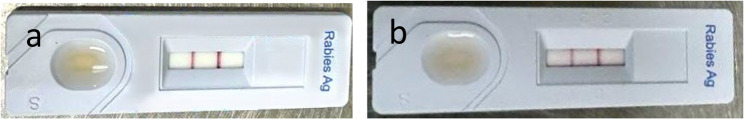
The rapid immunochromatographic diagnostic test (RIDT) showing a positive result for both goats (**A**) Case 1 and (**B**) Case 2. The test result is considered positive on the appearance of two purple colour bands (’’C’’) and test (’’T’’) within the test window. It is negative when only a single purple band (’’C’’) appears within the test window. The test is considered invalid when the band at the control does not appear

The goat brain sample was collected post-mortem and tested positive for RABV using a rapid immunochromatographic diagnostic test (RIDT) on 21 st September 2023 (Fig. 2). The goat’s brain samples were collected using the straw method via the occipital foramen route, as described in the World Organisation for Animal Health (WOAH) Terrestrial Manual, Chap. 3.1.19 “Rabies (Infection with Rabies Virus and Other Lyssaviruses)”, Sect. 1.1.1. “Occipital foramen route for brain sampling” [5, 6]. The brain sample was sent to the Sarawak Veterinary Diagnostic Laboratory (SVDL) for confirmation, using the Direct Fluorescent Antibody Test (DFAT) on 26 September 2023. The SVDL is under the Department of Veterinary Services Sarawak, a government agency responsible for disease surveillance and diagnostics for livestock and companion animals in Sarawak, Malaysia. Consequently, a temporary closure notice and movement restriction order were immediately issued to the farm. All remaining goats were vaccinated against rabies, any showing clinical signs or abnormal behaviour were isolated, and the entire herd was placed under a one-month quarantine in accordance with the Department of Veterinary Services Malaysia standard operating procedure [7] for animals exposed to suspected rabid cases.

The farm owner’s suspected rabid dog died on 20th September 2023, approximately three days after it bit (17th September 2023) the goat. The dog’s carcass was buried directly, unwrapped, in an unmarked spot by the farmer. The dog’s remains were exhumed from the burial site on 10th October 2023, about 20 days after death. The carcass was significantly decomposed and mixed with moist soil and wood pieces, excluding the possibility of obtaining reliable diagnostic test results for this study.

An investigation was conducted to determine the potential risk of rabies transmission following the incident. Findings confirmed that the dog had not bitten any other individuals, pets, or livestock within the neighbourhood. Nevertheless, the farm owner and workers were advised to undergo, and subsequently received, post-exposure prophylaxis (PEP) in accordance with the rabies control guidelines of the Ministry of Health Malaysia and the Department of Veterinary Services [8].

### Case 2: SVDL 2000/23

After the initial incident, the Bintulu Veterinary officer revisited the farm on the 26th of September 2023 to administer rabies vaccinations to a total of 21 heads of goats as part of a comprehensive disease control effort. However, during the visit, another 1-year-old female goat (a Boer cross) on the farm was found to show similar neurological clinical signs. Clinical signs observed in the new case included total recumbency, excessive drooling, stiffness, pedalling movements, tachycardia (120 bpm), dehydration, and episodes of opisthotonos (Fig. 1). These signs were consistent with rabies infection and closely resembled those observed in the first infected goat on this farm. This goat had also been exposed to the same suspected rabid dog that had bitten Case 1, suggesting that the dog was the probable source of infection rather than direct transmission from Case 1. While goat-to-goat transmission cannot be entirely excluded, such events are considered highly unlikely and are not supported by current evidence in the literature. The goat died from choking after a veterinary officer attempted to give it water to test its swallowing reflex. The goat brain sample was collected post-mortem and tested using the RIDT on 26th September 2023, which yielded a positive result (Fig. 2). The brain sample was submitted to the SVDL for further confirmation by DFAT.

### Laboratory investigation

#### RIDT and DFAT

The brain samples were initially tested at the farm premises using a RIDT assay (Fig. 2) as a rapid field screening test for rabies antigen detection (BioNote, Inc. 2–9, Seogu‐dong, Hwaseong‐si, Gyeonggi‐do, Korea), according to the manufacturer’s instructions. According to Tenzin et al., the rapid test kit showed a sensitivity of 92% (95% CI: 85.9–95.6) and specificity of 100% (95% CI: 93.4–100) using DFAT as the reference standard [9]. The samples were then sent to the SVDL for the DFAT testing. No ethical approval was required, as the SVDL is legally authorised to collect and test samples from animals suspected of rabies under the Sarawak Law, specifically Chap. 32, VPHO 1999, Sect. 38 (4) and 39 (1).

**Fig. 3:**
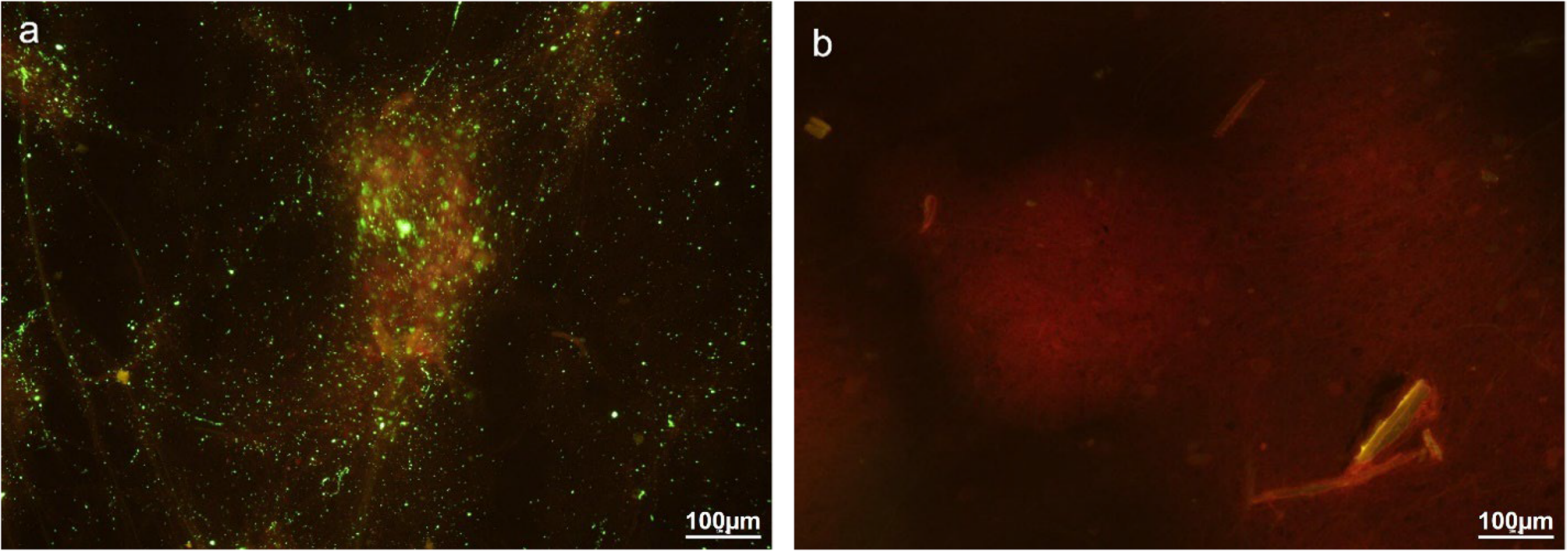
Results of DFAT from the brain of Case 1 with suspected rabies under 200X magnification. **a** Suspected goat brain sample, showing strong and diffuse positive signals of bright apple green fluorescence indicating the presence of rabies antigen; (**b**) control normal goat brain sample, in which no bright apple green fluorescence of rabies antigen was observed

**Fig. 4:**
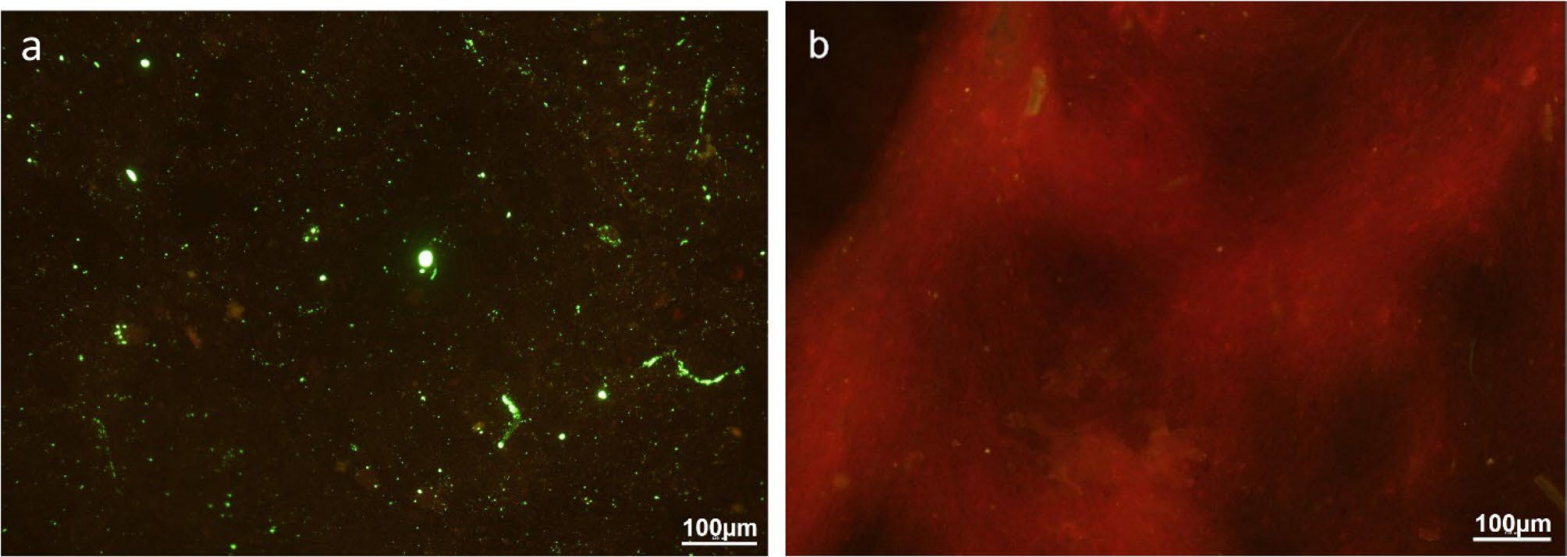
Results of DFAT from the brain of Case 2 with suspected rabies under 200X magnification. **a** Suspected goat brain sample, showing strong and diffuse positive signals of bright apple green fluorescence indicating the presence of rabies antigen; (**b**) control normal goat brain sample, in which no bright apple green fluorescence of rabies antigen was observed

The DFAT test was performed using FITC Anti-Rabies Monoclonal Globulin (Fujirebio Diagnostics, Inc., Japan), following the procedure described in WOAH Manual of Diagnostic Tests and Vaccines for Terrestrial Animals, Chap. 3.1.19 “Rabies (Infection with Rabies Virus and Other Lyssaviruses),” Sect. 1.3.1 “Immunochemical identification of rabies virus antigen” [6]. In brief, a small portion of the brain sample was smeared onto a glass slide using a wooden stick, then pressed against a wooden tongue depressor to ensure an even, thin smear before being air-dried. The smear was then fixed in cold acetone at − 20 °C for at least 30 min. After air-drying, 50 µl of rabies antibody conjugate (depending on smear size) was applied to cover the smear and incubated at 37 °C for 30 min in a humid chamber. Excess conjugate was removed by rinsing the slides twice with Phosphate-Buffered Saline (PBS) solution (pH 7.4) and once with distilled water for 3–5 min, followed by air drying. Coverslips were then mounted using Dako mounting medium, and the slides were examined under a fluorescent microscope (Nikon, Germany) within two hours of staining. A positive result was indicated by the presence of bright apple-green fluorescence against a brick-red background, while the absence of apple-green fluorescence indicated a negative result. The positive and negative control slides were included in the DFAT to ensure result validity. Both RIDT (Fig. 2) and DFAT analyses (Figs. 3 and 4) confirmed the presence of RABV in the brain tissues of both goats. All necropsy and confirmatory testing were conducted under SVDL biosafety procedures consistent with WOAH guidance.

### Sample processing and nucleic acid extraction

Brain tissue samples obtained from Case 1 and Case 2 were homogenized in 1x PBS using a Fastprep-24 5G instrument with Lysing Matrix D (MP Biomedicals) according to the manufacturer’s instructions. The homogenates were then syringe filtered through 0.2 μm Millipore filter (Merck Millipore) prior to nucleic acid extraction. Column-based extraction was performed using High Pure Viral Nucleic Acid kit (Roche) following the manufacturer’s protocol. Viral RNA was eluted from the column in 50 µL of RNA Storage Solution (Thermo Fisher Scientific).

### PCR-Tiling amplification and nanopore sequencing

To obtain comprehensive PCR coverage of the RABV genome, we designed an in-house set of overlapping primers based on the closest known whole-genome Indonesian RABV isolate from the SEA1a lineage, available in GenBank (Accession No.: KX148266) [10]. Primer design was performed using OLIGO 7 [11], and the primers were synthesized in two separate pools. Non-uniform amplicon coverage was expected due to primers targeting conserved regions with inconsistent spacing, resulting in amplicons ranging from 900 bp to 1300 bp in length.

A PCR-tiling approach was integrated into the sequencing workflow to increase sensitivity and to improve sequencing yields. cDNA synthesis was performed using SuperScript™ IV Reverse Transcriptase (Thermo Fisher Scientific) following the manufacturer’s protocol in a 10 µL reaction volume. The cDNA served as a template for amplification using Q5^®^ High-Fidelity DNA Polymerase (New England Biolabs) in a 20 µL reaction volume under the following PCR conditions for 32 cycles: 98 °C for 30 s (denaturation), 60 °C for 20 s (annealing), and 72 °C for 4 min (elongation). Each reaction was quantified using the Qubit™ dsDNA High Sensitivity (HS) Assay Kit on a Qubit Fluorometer.

Library preparation for both RABV samples was performed using the Native Barcoding Kit V14 (SQK-NBD114.96) and sequenced on a MinION device with FLO-MIN114 (R10.4.1) flow cell from Oxford Nanopore Technologies [12]. The raw data was basecalled using Guppy v5.0.16, and the demultiplexed, barcode-trimmed FASTQ files were analysed using the interARTIC v0.4.4 workflow [13], locally configured with an in-house RABV primer scheme. Each sample contains >17,000 pass read count with min length of above 400 bp, read quality mean of above 16 and mean read coverage of all 13 amplicons covering the 11,932 bp genome above 250. Primer sites were trimmed and the final consensus was generated using Medaka pipeline v2.1.0 as part of the interARTIC workflow where reads were polished using model “r941 min high g360” and aligned to a known reference genome (KX148266) with a minimum read depth of 20 to mask low-confidence regions. Both RABV samples shared 100% sequence identity for the complete nucleoprotein (N) gene region. The consensus RABV N gene sequences obtained from the two goat cases in this study were deposited in GenBank under the accession numbers PX112048 (Case 1) and PX112049 (Case 2).

### Phylogenetic analysis

To determine the phylogenetic relationships of our two near-complete RABV genomes, we included publicly available RABV sequences from the surrounding region and representative global genotypes [10, 14–18]. All selected sequences, including those derived from our isolates, were aligned using MAFFT version 7.0 [19]. The majority of national and regional comparator sequences in GenBank were limited to partial or complete N gene. To maximize the inclusion of epidemiologically important strains, phylogenetic analysis was restricted to a conserved 496 bp fragment of the N gene (positions 39–534, based on the reference sequence KX148266.1). We acknowledge that using a short fragment limits phylogenetic resolution and may obscure finer-scale genetic differences between closely related strains.

A maximum-likelihood phylogenetic tree with 1,000 bootstrap replications was constructed using the Molecular Evolutionary Genetics Analysis (MEGA, version 12.0.11) program [20], applying the T92 + G + I (Tamura 3-parameter substitution model) [21]. The model was selected using MEGA’s inbuilt “Find Best DNA/Protein Models (ML)” function, which evaluated candidate models based on the Bayesian Information Criterion (BIC).

**Fig. 5:**
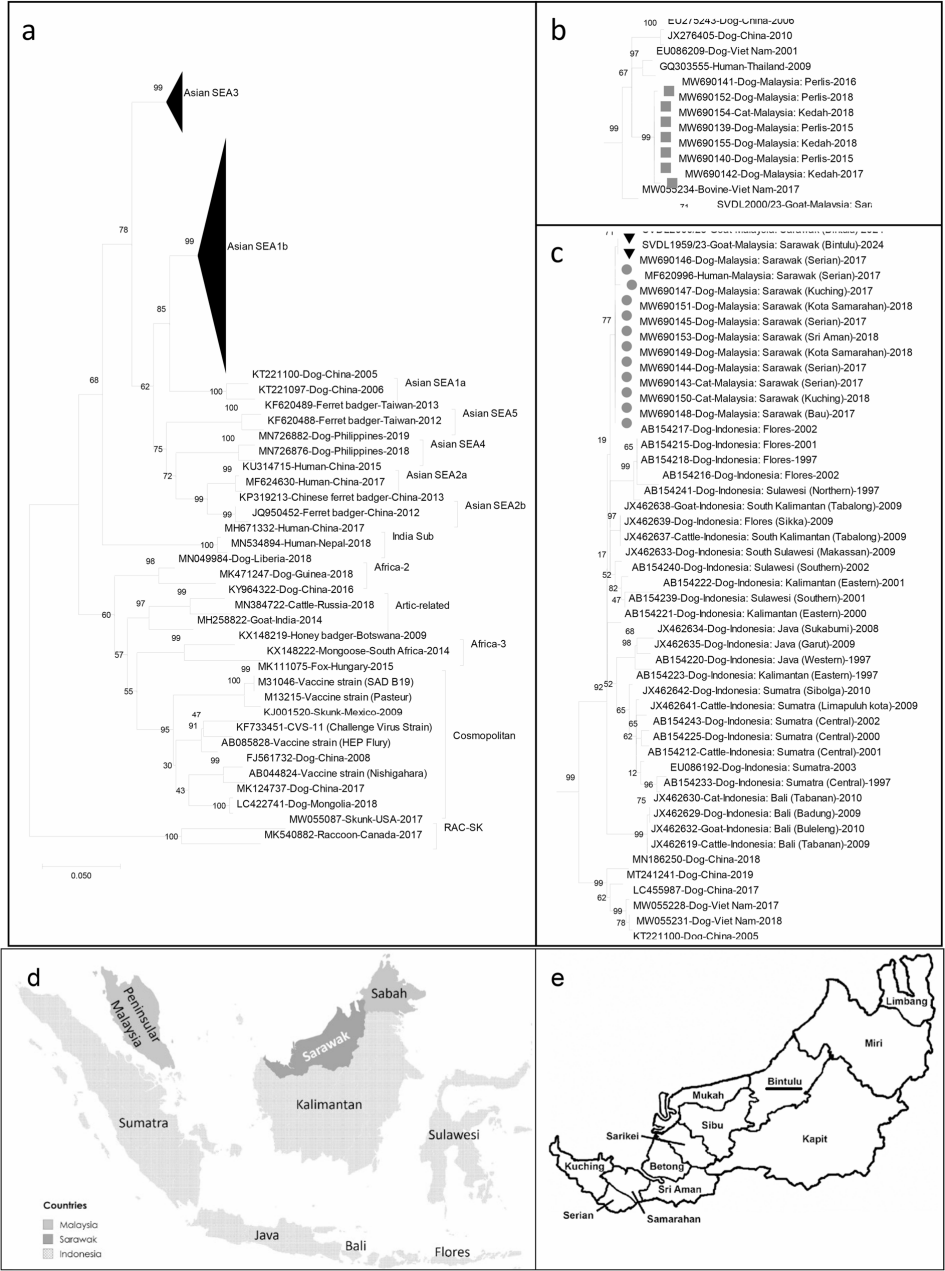
Phylogenetic tree of 20 Malaysian and 69 reference RABVs based on partial N gene of RABV: (**a**) full phylogenetic tree; (**b**) expanded Asian SEA3 subclade; (**c**) expanded Asian SEA1b subclade; (**d**) map of Malaysian and Indonesian regions around Sarawak; (**e**) map of Sarawak divisions [22], with Bintulu underlined. There were a total of 496 positions in the final dataset as all nucleotide positions with less than 95% site coverage were eliminated. Tree was constructed using MEGA version 12.0.11 by Maximum-likelihood statistical method and 1,000 bootstrap replicates. RAC-SK RABVs were used as outgroup. Black triangles (▼) represent the Sarawak goat RABVs. Grey circles () represent other Sarawak RABVs, grey squares () represent Peninsula Malaysian RABVs. Each RABV strain name is suffixed by GenBank accession number/host animal/country/year. Phylogenetic tree clades and subclades are labeled with a bracket and name. Map (**d**) was created with MapChart.net

Phylogenetic analysis of the partial N gene of the RABV showed that the two goat rabies sequences were highly similar to previously reported RABV strains from the Asian clade SEA1b subclade identified in Sarawak from 2017 to 2018. In contrast, published sequences from Peninsular Malaysia cluster within the Asian clade SEA3 subclade, indicating that Sarawak and Peninsular Malaysia harbor distinct rabies virus lineages (Fig. 5). Comparing the complete N gene region (1,353 bp) of the two goat sequences to the previously reported Sarawak strains, the calculated percentage of similarity ranged between 99.5% and 99.8%. To further illustrate this relationship, we constructed a supplementary phylogenetic tree based on the complete N gene for a subset of Asian SEA1b subclade sequences, which confirmed that the two goat-derived sequences clustered closely with earlier Sarawak strains (Supplementary Fig. S1). This finding suggests the persistent circulation of the same viral lineage in the region. Furthermore, the Sarawak RABV strains were also closely related to strains from the Kalimantan, Flores, and Sulawesi regions of Indonesia (Fig. 5).

## Discussion and conclusion

In this report, we document the first two laboratory-confirmed cases of rabies in goats in Sarawak, Malaysia. A dog on the same farm showed clinical signs consistent with rabies; however, laboratory confirmation was not obtained. The presence of clinically suspected rabies in a dog alongside confirmed cases in goats raises strong suspicion of local virus circulation on the premises. This suggests that rabies transmission may have been occurring prior to detection and highlights the potential for under-recognised spillover into livestock species, which are not typically included in routine rabies surveillance in this region.

From Case 2, an important practical note emerges: testing the swallowing reflex with water in encephalitic livestock carries a significant risk of aspiration and sudden death. Clinicians should recognize this risk and assess the necessity of conducting the procedure, balancing clinical value against the likelihood of harm.

Sarawak, the largest state in Malaysia, located in the northwestern part of Borneo, has experienced widespread rabies outbreaks since 2017, with confirmed human and animal cases reported across several divisions, including Serian, Kuching, Samarahan, Sri Aman, Sibu, and Bintulu [23, 24]. While most reported animal cases involve dogs and, to a lesser extent, cats, these goat cases represent a significant expansion in the host range and geographic distribution of rabies in the state. These widespread cases underscore ongoing viral circulation and the risk of underreporting, especially among livestock and wildlife species.

The presence of rabies in goats is not unprecedented and should be recognised as a potential risk, particularly in regions where the disease is endemic. Rabies infections in goats have been reported in various countries, including Ghana, Mongolia and Brazil, often following bites from rabid dogs or wild carnivores [25–27]. Affected animals typically presented with neurological signs such as restlessness, excessive salivation, ataxia, aggression, vocalisation, and eventual recumbency. These clinical features were consistent with those observed in our cases. In those previous reports, laboratory confirmation was primarily achieved through the direct fluorescent antibody test (DFAT), histopathological evaluation, or Real time-PCR (RT-PCR). Indonesia had also reported 2 sequence-confirmed goat rabies cases, one each from Tabalong, South Kalimantan and Buleleng, Bali in 2009 [12] respectively, indicating the spillover from canine reservoirs to other domestic animals has already been happening in nearby regions for some time.

In this study, rabies diagnosis in both goats were confirmed through the post-mortem testing of their brain tissues by using the RIDT [9] and DFAT [10, 28] assays. The domestic dog suspected to be the reservoir in this study died and was buried before any diagnostic samples could be collected. When the incident was later reported, the remains had already undergone extensive decomposition and were intermixed with moist soil and wooden debris in a warm environment. An attempt was made to detect RABV RNA in the decomposed sample using an in-house real-time PCR assay; however, the result was negative, likely due to RNA degradation caused by the advanced state of decomposition. Although direct laboratory confirmation for the dog was unavailable, the evidence from the case history, clinical presentation in the goats and the dog exhibited clinical signs consistent with rabies, such as aggression and excessive salivation, before dying shortly after, as described by the farmers, strongly suggests that the dog was rabid and transmitted the RABV to the goat. The 100% N gene sequence similarity between the two goat samples suggests that both animals were infected with the same RABV strain, most plausibly through independent exposure to the rabid dog. While goat-to-goat transmission cannot be entirely excluded, such events are highly unlikely and are not supported by current evidence. Therefore, the precise source of infection for the second goat cannot be definitively established, and this remains a limitation of this report.

Additionally, the goat-derived sequences were firmly placed within the established Malaysian Sarawak RABV strains, indicating a sustained transmission cycle maintained by dogs. Their phylogenetic position, distinct from the lineage circulating in Peninsular Malaysia, indicates that these cases did not result from introductions from Peninsular Malaysia but rather from the persistence of the Sarawak strain. The clustering of the 2023 goat RABV sequences with strains from 2017 to 2018 suggests the continued local circulation of a specific RABV strains, highlighting potential gaps in current vaccination efforts and dog population management strategies. The close genetic similarities between the Sarawak RABV strain and Kalimantan RABV strain aligns with previous studies that have suggested cross-border transmission of rabies between these regions [13]. Phylogenetic analysis in this study indicates that the RABV strains detected in Sarawak are closely related to those from Kalimantan and Flores, Indonesia, suggesting a likely Indonesian origin of the outbreak, followed by sporadic internal transmission within Sarawak. However, this interpretation remains tentative due to limitations such as the small sample size, absence of confirmed dog samples, potential goat-to-goat transmission, and targeted sampling from affected farms.

Even though the goat rabies strain is genetically similar to the previous Sarawak strains, the risk of introducing new RABV strains from neighbouring countries remains a crucial concern. As rabies is a zoonotic disease primarily spread through the movement of infected animals, different circulating strains can be introduced from neighbouring countries via transboundary transmission. In Sarawak, the Immune Belt Enforcement Team (IBET) conducts targeted dog vaccination campaigns along border areas to reduce the risk of virus introduction from neighbouring countries, although up-to-date information on the rabies status in adjacent regions remains limited [29]. 

Sarawak supports diverse livestock farming, including pigs, poultry, cattle, goats, and buffaloes. This sector is vital for food security, rural livelihoods, and local employment. Farming in Sarawak is common in rural and semi-urban areas, often on a small to medium scale using a mix of traditional and semi-modern practices. However, smallholders frequently face constraints such as inadequate facilities, limited technical expertise, and scarce resources for animal health and disease prevention. Biosafety standards vary considerably between farms. The dog population dynamics in Sarawak adds another challenge, with both owned and free-roaming dogs found in rural villages and on town outskirts. While many households keep dogs for security or companionship, breeding is often informal and unregulated. Close contact between dogs and livestock increases the risk of disease transmission, particularly rabies, threatening both animal health and farmers’ livelihoods.

This study highlights the need to strengthen rabies surveillance by raising awareness of the disease in atypical hosts such as goats and equipping field teams with diagnostic tools for early detection across species. Effective rabies control requires an integrated approach combining early case identification, mass vaccination, and targeted public education. In response to the outbreak, the Sarawak state government has implemented measures including dog population control, mandatory dog licensing, and extensive vaccination programs for domestic dogs and cats, prioritizing high-risk areas. The Department of Veterinary Services Sarawak (DVSS) conducts free annual vaccination campaigns and public education initiatives through schools, community outreach, and media to promote responsible pet ownership, encourage prompt reporting of suspected cases, and improve safe animal handling practices [23]. These efforts have reduced confirmed human rabies cases in recent years, though gaps remain in remote farming communities [24]. This case underscores the need to expand vaccination coverage, improve access in rural areas, and enhance farmer and veterinary awareness of rabies risks in both companion animals and livestock. A sustained, adaptive strategy integrating surveillance, vaccination, and community engagement is essential to protect animal and human health.

## Supplementary Information

Supplementary Material 1.

## Data Availability

All data generated or analysed during this study are included in this published article. The consensus sequences have been deposited in GenBank under accession numbers PX112048 and PX112049.

## Abbreviations

RABV: Rabies Virus
N gene: Nucleoprotein gene
RIDT: Rapid Immunochromatographic Diagnostic Test
DFAT: Direct Fluorescent Antibody Test
NGS: Next Generation Sequencing
SVDL: Sarawak Veterinary Diagnostic Laboratory
WOAH: World Organisation for Animal Health, PBS-Phosphate Buffered Solution
