# Supplementary material for: First detection of rabies virus in encephalitic goats (Capra hircus) from Sarawak, Malaysian Borneo: a case study report
Source: BMC Vet Res. 2025 Nov 6;21:648. doi: 10.1186/s12917-025-05110-2 (PMC12590585; doi:10.1186/s12917-025-05110-2)
Supplement: Supplementary file 1 — Supplementary Material 1. [file 12917_2025_5110_MOESM1_ESM.docx]

**First detection of rabies virus in Encephalitic Goats (*Capra hircus*) from Sarawak, Malaysian Borneo: A case study report**

**Supplementary Information**

| **Case ID** | **Case 1 (SVDL 1959/23)** | **Case 2 (SVDL 2000/23)** |
| --- | --- | --- |
| **Age/sex/breed** | 1-year-old / F / local mix | 1-year-old / F / Boer cross |
| **Exposure history** | Suspected rabid dog (mixed with herd); bitten on nose,  17^th^ Sept 2023 | Contact with same suspected rabid dog and Case 1,  no known bite history |
| **Symptom onset** | 19^th^ Sept 2023 | On or before 26^th^ Sept 2023 |
| **Key clinical signs** | Aggression, hypersalivation, excessive bleating, recumbency, dehydration, stiffness, pedalling | Hypersalivation, opisthotonos, tachycardia, recumbency, dehydration, stiffness, pedalling |
| **Date of death** | 21 Sept 2023 (died naturally ~6 hours after recumbency) | 26 Sept 2023 (choked during swallowing reflex test) |
| **Farm actions** | Farm closure & movement restriction; herd vaccinated; suspect goats isolated;  1-month quarantine | Farm quarantine period maintained following Case 2 detection |
| **Diagnostic confirmation** | Post-mortem brain tested RIDT+, confirmed DFAT+ | Post-mortem brain tested RIDT+, confirmed DFAT+ |

**Supplementary Table S1. Summary of clinical and epidemiological features of the two laboratory-confirmed goat rabies cases in Bintulu, Sarawak, 2023.**

**Supplementary Fig. S1. Phylogenetic tree of the two Bintulu goat RABVs, ten other Sarawak RABVs and six reference RABVs based on the complete N gene.** Only SEA1b subclade sequences with complete N gene coverage were included, with positions containing gaps or missing data removed by complete deletion, resulting in 1,353 positions in the final dataset. The tree was constructed using the maximum-likelihood method with 1,000 bootstrap replicates in MEGA version 12.0.11 [20]. The Pasteur vaccine strain (Cosmopolitan clade; GenBank accession M13215) was used as the outgroup.

Black triangles (▼) denote the Sarawak goat RABV sequences, and grey circles () denote other Sarawak RABV sequences. Each strain is labeled with GenBank accession number/host animal/country/year.

This maximum-likelihood phylogenetic tree was constructed applying the T92+I (Tamura 3-parameter substitution model) [21]. The model was selected using MEGA's inbuilt "Find Best DNA/Protein Models (ML)" function, which evaluated candidate models based on the Bayesian Information Criterion (BIC).
